# Serum levels of trimethylamine N-oxide and kynurenine novel biomarkers are associated with adult metabolic syndrome and its components: a case-control study from the TEC cohort

**DOI:** 10.3389/fnut.2024.1326782

**Published:** 2024-01-23

**Authors:** Atieh Mirzababaei, Maryam Mahmoodi, Abbasali Keshtkar, Haleh Ashraf, Faezeh Abaj, Neda Soveid, Mahya Mehri Hajmir, Mina Radmehr, Pardis Khalili, Khadijeh Mirzaei

**Affiliations:** ^1^Department of Community Nutrition, School of Nutritional Sciences and Dietetics, Tehran University of Medical Sciences, Tehran, Iran; ^2^Department of Cellular and Molecular Nutrition, School of Nutritional Science and Dietetics, Tehran University of Medical Sciences, Tehran, Iran; ^3^Department of Disaster and Emergency Health, School of Public Health, Tehran University of Medical Sciences, Tehran, Iran; ^4^Cardiac Primary Prevention Research Center, Cardiovascular Diseases Research Institute, Tehran University of Medical Sciences, Tehran, Iran; ^5^Department of Nutrition, Dietetics and Food, School of Clinical Sciences at Monash Health, Monash University, Clayton, VIC, Australia; ^6^Department of Nutrition, Science and Research Branch, Islamic Azad University, Tehran, Iran

**Keywords:** kynurenine, trimethylamine N-oxide, metabolic syndrome, dysbiosis 2, gut microbiota metabolites

## Abstract

**Background:**

Epidemiologic research suggests that gut microbiota alteration (dysbiosis) may play a role in the pathogenesis of metabolic syndrome (MetS). Dysbiosis can influence Trimethylamine N-oxide (TMAO) a gut microbiota-derived metabolite, as well as kynurenine pathways (KP), which are known as a new marker for an early predictor of chronic diseases. Hence, the current study aimed to investigate the association between KYN and TMAO with MetS and its components.

**Methods:**

This case-control study was conducted on 250 adults aged 18 years or over of Tehran University of Medical Sciences (TUMS) Employee’s Cohort study (TEC) in the baseline phase. Data on the dietary intakes were collected using a validated dish-based food frequency questionnaire (FFQ) and dietary intakes of nitrite and nitrate were estimated using FFQ with 144 items. MetS was defined according to the NCEP ATP criteria. Serum profiles TMAO and KYN were measured by standard protocol.

**Result:**

The mean level of TMAO and KYN in subjects with MetS was 51.49 pg/mL and 417.56 nmol/l. High levels of TMAO (≥30.39 pg/mL) with MetS were directly correlated, after adjusting for confounding factors, the odds of MetS in individuals 2.37 times increased (OR: 2.37, 95% CI: 1.31–4.28, *P*-value = 0.004), also, high levels of KYN (≥297.18 nmol/L) increased odds of Mets+ 1.48 times, which is statistically significant (OR: 1.48, 95% CI: 0.83–2.63, *P*-value = 0.04). High levels of TMAO compared with the reference group increased the odds of hypertriglyceridemia and low HDL in crude and adjusted models (*P* < 0.05). Additionally, there was a statistically significant high level of KYN increased odds of abdominal obesity (*P* < 0.05).

**Conclusion:**

Our study revealed a positive association between serum TMAO and KYN levels and MetS and some of its components. For underlying mechanisms and possible clinical implications of the differences. Prospective studies in healthy individuals are necessary.

## Introduction

The World Health Organization (WHO) defines metabolic syndrome (MetS) as a pathological state consisting of obesity, insulin resistance, high blood pressure, elevated lipid levels, and waist-to-hip ratio (WHR); MetS occurs if at least three of the above-mentioned parameters are observed (1). It is estimated that 34% of Americans suffer from metabolic syndrome (2). Based on data from the Tehran Lipid and Glucose Study (TLGS), the prevalence of metabolic syndrome in adult adolescents in Iran was 24% in women and 42% in men (3). In addition, the whole incidence of metabolic syndrome in individuals 20 years of age and older was found to be 550.9 per 10,000; for men, it was 794.2 per 10,000, and for women, it was 443.5 per 10,000 (4). Numerous variables, such as chronic inflammation, autonomic disorders, and oxidative stress, are linked to the etiology of MetS (5). Several lines of recent scientific research suggest that alterations in the gut microbiota play a crucial role in the pathogenesis of MetS (6, 7). With the extensive westernization of lifestyle, changing the gut microbiota composition (dysbiosis) and its metabolites such as Trimethylamine N-oxide (TMAO), as well as affecting cellular pathways like the Tryptophan (TRP) pathway to produce kynurenine (KYN), has received more attention than previously due to their possible roles as an early predictor of cardiovascular disease (CVD) and MetS (8–10).

Trimethylamine N-oxide is an organic compound, gut microbiota-derived metabolite, which has recently been found as a new potentially important reason for increased atherosclerosis and MetS (11). Of all the pro-atherosclerotic mechanisms postulated for TMAO the most important are, a rise in vascular inflammation, the platelet hyper-responsiveness, the blockage of reverse cholesterol transport, reducing high-density lipoprotein cholesterol (HDL) in the liver, and affecting bile acids metabolism (12, 13). So TMAO may promote dyslipidemia via regulating cholesterol balance (14). Several systematic reviews and meta-analysis studies found a dose-dependent relationship between circulating levels of TMAO and elevated cardiovascular risk and mortality in humans (15–18). However, there is controversy about the specific role of TMAO in the pathogenesis of MetS, as a collection of cardiometabolic risk factors consisting of abdominal obesity, disrupted lipid profile, high blood sugar, and hypertension which predispose individuals to CVD (11). The kynurenine pathway (KP), which is known as the TRP degradation pathway, can be influenced by the gut microbiota (19). TRP is an exogenous amino acid that is metabolized to KYN by indoleamine 2, 3-dioxygenase (IDO) (20). Inflammatory factors like LPS-derived pathogenesis microorganisms or interleukin (IL-6) can induce IDO activation (21). KP appears to be involved in the development of many chronic inflammatory metabolic disorders, including Mets, and atherosclerosis, all of which are commonly recognized as risk factors for CVD (22–24). TRP degradation via KP has been reported to increase in patients with ischemic heart disease and atherosclerotic lesions (23). Furthermore, it has been shown that elevated IDO activity and KYN are positively correlated with overweight and dyslipidemia (23, 25). However, studies on the relationship between the KYN pathway and dyslipidemia are still ambiguous and contradictory. For example, in an experimental study on IDO-deficient mice, an increase in TG levels was observed along with a decrease in HDL (26), while in another animal study, it was observed that one of the downstream metabolites of the KP (3-hydroxy anthranilic acid) by decreasing hepatic fat accumulation, lowered plasma levels of triglyceride (TG) and cholesterol and improving atherosclerosis in LDLR^–/–^ mice (27).

Altogether, the literature suggests a connection between the KP pathway and TMAO and some components of MetS. However, most of these data derive from experimental research in animals, and little is known about its relevance in human cohorts. Further research needs to clarify the pathway and cellular-molecular mechanism in this context which may lead one to consider whether plasma KYN and TMAO can be a new risk factor for predicting MetS in predisposed persons. So, the primary aim of the current study is the measurement of gut microbiota metabolites such as KYN and TMAO and the main goal is to investigate the mentioned metabolites association with MetS and its components in adults.

## Materials and methods

### Study population and research design

The design of a recent study is case-control that was carried out from 2018 to 2020 on a subsample of subjects ages ranging from 20 to 50 years and participated in the Tehran University of Medical Sciences (TUMS) Employee’s Cohort study (TEC). The Ethics Committee of the TUMS evaluated and approved the study protocol (IR.TUMS.MEDICINE.REC.1401.064) and all participants filled out consent at the beginning (28). Subjects free of any acute or chronic disease history, such as polycystic ovary syndrome (PCOS), cancers, diabetes mellitus, hepatic, intestinal disorder, infectious diseases, and, kidney disease entered the study. Adherence to specific/unusual dietary intake, alcohol consumption, significant body weight changes, pregnancy, and lactation, during the last year, were considered also as exclusion criteria. In addition, subjects taking medications that influence body weight and/or glucose and/or lipid-lowering drugs and probiotics were not eligible to take part in the current study. Also, participants with energy intake of less than 800 or more than 4200 (kcal/day) were excluded. Eligible Participants have been categorized into two separate groups; the first group included 125 MetS subjects based on National Cholesterol Education Program Adult Treatment Panel III (NCEP ATP III) definition and the other group consisted of 125 non-MetS subjects based on a random number table of subjects selected.

### Sample size

The sample size was computed according to the following formula:

$$N=\left[{\left({\text{Z}}_{1-\alpha }+{\text{Z}}_{1-\beta }\right)}^{2}\,\left({\text{SD}}_{1}^{2}+{\mathrm{SD}}_{2}^{2}\right)\right]/\Delta^{2}$$

β = 20%, and α = 0.05 Then, with 95% confidence and 80% power (29, 30), 125 cases and 125 control aged 20–50 years were required in this case-control study.

### Dietary intake measurement

A self-administered, 144-item food frequency questionnaire (FFQ) that has been previously validated was used to collect usual dietary intake information (31). The FFQ covered the following items: mixed dishes (cooked or canned), dairy products (dairy, butter, and cream), grains (different types of bread, cakes, biscuits, and potato), vegetables, fruits, miscellaneous food items, and beverages (including sweets, fast foods, nuts, desserts, and beverages). To estimate the quantity of food intake common portion sizes were considered. Participants’ food frequency intakes were reported using nine multiple-choice response categories, ranging from “never or less than once a month” to “6 or more times per day.” Utilizing the national nutrient databank of the US Department of Agriculture (USDA), daily nutrient intakes were determined. At last, obtained data were analyzed by the NUTRITIONIST 4 (First Data Bank, San Bruno, CA, USA) food analyzer (32).

### Biochemical indicators assessment

All participants’ blood samples were collected between 8:00 and 9:30 AM after 12 to 14 h of overnight fasting. Then the blood samples were centrifuged for 10 min at 3000 rpm to obtain the plasma, were aliquoted to microtubes, and immediately stored for further analysis. Fasting blood sugars (FBS), low-density lipoprotein (LDL-C), Total cholesterol (TC), TG, and HDL-C were measured via enzymatic colorimetric method and phosphor tungstic acid. Serum concentrations of KYN and TMAO were measured using enzymatic methods. Analyses were carried out using available commercial kits (Shanghai Crystal Day Biotech, Shanghai, China).

### Assessment of blood pressure

We measured systolic blood pressure (SBP), and diastolic blood pressure (DBP), three times from the right hand and in a sitting position, using a standardized mercury sphygmomanometer, at time intervals of 20 min, 2, and 4 h after admission, and we reported the average of these 3 measurements for the subjects.

### Anthropometric measurements

To measure each subject’s body weight, a calibrated digital scale was used to the nearest 0.1 kg when they were wearing light clothes without shoes. For measuring height, non-elastic tape, accurate to approximately 0.1 cm was used in a normal standing position with barefoot. Waist circumference (WC) was assessed, using a soft tape meter with a precision of 0.1 cm at the end of a normal exhale from the narrowest part of the waist. The hip circumference (HC) was estimated by placing an elastic measuring tape with 0.1 cm precision on the most noticeable, marked area of the buttocks. By dividing WC by HC, WH) was calculated to compute body mass index (BMI), using the formula weight/height^2^ (kg/m^2^).

### Mets and the definition of its components

National cholesterol education program ATP III suggests diagnostic criteria for defining MetS. Having at least 3 of the following metabolic abnormalities was considered as MetS based on NCEP ATP III definition (33, 34): (1) Hyperglycemia as FBS ≥ 100 mg/dL (5.6 mmol/L), (2) Hypertriglyceridemia as serum TG ≥ 150 mg/dL (1.69 mmol/L), (3) Low HDL-C as serum HDL-C < 40 mg/dl (1.03 mmol/l) in men and < 50 mg/dl (1.29 mmol/l) in women, (4) Hypertension as BP ≥ 130/85 mmHg, and (5) Abdominal obesity as WC > 90 cm in men and >80 cm in women. However, according to NCEP ATP III, modified for the Iranian population, abdominal obesity was considered as WC > 95 cm.

### Other variables measurements

Based on the validated and reliable self-report International Physical Activity Questionnaire (IPAQ), physical activity (PA) was assessed (35). With this instrument PA level in the last week was estimated and the output data was presented as metabolic equivalent (MET). Due to the frequency and time spent, the intensity of the PA level was determined. Finally, scores were computed and PA was categorized into light, moderate, high, and very high-intensity activities, then total MET-min/week was obtained by adding up the scores of various activities. Furthermore, the other variables such as occupation, education, marital, and social socioeconomic status (SES) were assessed via a self-report socio-demographic questionnaire.

### Statistical analysis

For checking data normality, the Kolmogorov-Smirnov test was employed. MetS status was separated into two groups based on NCEP ATPIII criteria; patients with MetS and without MetS. To examine the relationship between qualitative variables in the case and control groups, the chi-square test for crude and the Cochran-Mantel-Hansel chi-square test with adjustments factors were used. For quantitative variables, the *t*-test and Mann-Whitney test were used. Binary logistic regression was used to estimate the crude and adjusted odds ratio (OR) with a 95% confidence interval (CI) for the odds of MetS. We considered age, sex, energy intake, BMI, and physical activity as potential confounders in the adjusted model. In all of the tests, *P*-value < 0.05 was considered significant. All statistical analyses were done using SPSS software version 25.

## Results

### Study population characteristics

The present study included 250 Iranian men and women. The mean values of age, weight, height, and BMI of participants were 41.27 (8.70) years, 76.60 (15.85) kg, 165.76 (9.16) cm, and 27.24 (4.48) kg/m^2^, respectively. A total of 26.8 and 73.2 percent of the participants were male and female, respectively. A total of 80.3% of the participants were married and 3.6% of them had moderate physical activity.

### Characteristics of the study population based on MetS

Characteristics of participants are presented based on MetS in Table 1. According to statistical analyses in two groups with and without MetS, in terms of anthropometric measurements, there was a significant difference between weight, height, WHR, WC, and HC participants (*P* < 0.05). So that the average of these measurements was higher in MetS+ than in individuals without MetS, which was statistically significant in both crude and adjusted models (age, energy intake, physical activity, and sex) (*P* < 0.05).

**TABLE 1:** Characteristics of study population based on MetS.

Variables	With MetS *n* = 125	Without MetS *n* = 125	*P*-value	*P*-value*
**Demographic**				
Age (year)	40.78 (8.58)	41.73 (8.82)	0.40	0.41
**Sex**				
Male	22 (32.8)	45 (67.2)	**0.001**	**0.001**
Female	103 (56.3)	80 (43.7)		
**Anthropometric**				
Weight (kg)	81.86 (15.85)	71.60 (12.88)	**<0.001**	**<0.001**
Height (cm)	167.08 (9.15)	164.51 (9.03)	**0.03**	**0.01**
BMI (kg/m^2^)	27.20 (4.40)	27.27 (4.57)	0.90	0.88
WHR	0.88 (0.08)	0.83 (0.08)	**<0.001**	**<0.001**
WC (cm)	95.19 (10.72)	86.08 (10.44)	**<0.001**	**<0.001**
HC (cm)	106.77 (9.39)	102.44 (6.71)	**<0.001**	**0.001**
**Blood parameters**				
TG (mg/dl)	205.91 (104.76)	108.18 (57.78)	**<0.001**	**<0.001**
HDL (mg/dl)	40.77 (6.13)	46.42 (9.43)	**<0.001**	**<0.001**
LDL (mg/dl)	107.50 (30.36)	103.95 (24.26)	0.32	0.79
TC (mg/dl)	193.12 (45.25)	175.59 (34.88)	**0.001**	**0.010**
FBS (mg/dl)	104.83 (47.08)	83.97 (9.99)	**<0.001**	**<0.001**
**Blood pressure**				
SBP (mmHg)	117.99 (20.13)	114.42 (14.29)	0.11	0.12
DBP (mmHg)	78.48 (13.23)	75.20 (9.85)	**0.03**	**0.02**
**Metabolites**				
TMAO (pg/mL)	51.49 (42.06)	37.50 (29.07)	**0.002**	**<0.001**
KYN (nmol/l)	417.56 (290.98)	369.52 (276.72)	0.18	0.20
**Qualitative variable**				
**Marriage status**				
Single	31 (61.2)	19 (38.8)	0.07	0.08
Married	94 (47)	106 (53)		
**Education level**				
≤Diploma	46 (51.2)	42 (48.8)	0.56	0.48
University educated	79 (48.8)	83 (51.2)		
**Job group**				
Office	60 (47.2)	67 (52.8)	0.97	0.91
Clinical	34 (50.7)	33 (49.3)		
Technical	21 (60)	14 (40)		
Security	10 (47.6)	11 (52.4)		
**Socio-economic status**				
Low	5 (50)	5 (50)	0.54	0.55
Moderate	115 (50.9)	111 (49.1)		
High	5 (35.7)	9 (64.3)		
**Physical activity**				
Low	119 (49.4)	122 (50.6)	0.30	0.31
Moderate	6 (66.7)	3 (33.3)		

BMI, body mass index; WHR, weight to hip ratio; WC, waist circumference; HC, hip circumference; TG, triglyceride; HDL, high-density lipoprotein; LDL, low-density lipoprotein; TC, total cholesterol; FBS, fasting blood sugar; SBP, systolic blood pressure; DBP, diastolic blood pressure; TMAO, trimethylamine N-Oxide; KYN, kynurenine. Quantitative variables as means (±SD) obtained from the independent *t*-test. Qualitative variables *N* (%) obtained from the chi-square analysis. *P*-value < 0.05 were considered significant, bolding in Table. *P*-value* for adjustment model, based on age, energy intake, physical activity, sex and BMI.

In addition, among the blood parameters, the average levels of TG, TC, and FBS in MetS+ were higher than those of MetS-, and this difference was statistically significant (*P* < 0.05).

The mean value of TMAO in MetS+ was 51.49 pg/mL and in MetS- was 37.50 pg/mL, which was significant in both crude (*P* = 0.002) and adjusted models (*P* < 0.001). Although the mean value of KYN was higher in MetS+ (417.56 nmol/l) than in MetS- (369.52 nmol/l), it was not statistically significant (*P* > 0.05).

### Dietary intake among subjects based on MetS

In Table 2, the dietary intake of the study participants based on MetS is presented. In the crude and adjusted model, there was no significant difference between the average intake of macronutrients and micronutrients of MetS+ and MetS- (*P* > 0.05).

**TABLE 2:** Dietary intake among subjects based on MetS.

Variables	With MetS *n* = 125	Without MetS *n* = 125	*P*-value	*P-*value*
**Macronutrient**				
Energy (kcal/day)	2656.77 (684.47)	2630.82 (714.45)	0.79	–
Cho (gr/day)	372.32 (103.33)	376.18 (105.99)	0.79	0.23
Fat (gr/day)	87.79 (30.89)	84.57 (27.80)	0.43	0.33
Protein (gr/day)	102.53 (28.42)	99.99 (29.53)	0.53	0.40
**Micronutrient**				
**Minerals**				
Ca (mg/day)	988.71 (400.83)	963.67 (337.74)	0.63	0.62
P (mg/day)	1202.81 (438.75)	1162.44 (395.34)	0.49	0.46
Mg (mg/day)	238.39 (76.31)	238.20 (79.51)	0.98	0.84
Fe (mg/day)	19.91 (6.03)	19.96 (6.09)	0.95	0.50
Zinc (mg/day)	7.84 (2.56)	7.63 (2.44)	0.56	0.53
Copper (mg/day)	1.19 (0.49)	1.20 (0.50)	0.88	0.64
Na (mg/day)	3211.45 (963.02)	3065.73 (976.69)	0.28	0.18
K (mg/day)	3008.71 (1107.95)	3005.57 (1086.20)	0.98	0.87
**Vitamins**				
A (IU/day)	1923.49 (2101.10)	1896.31 (1459.19)	0.91	0.90
D (μg/day)	1.60 (1.53)	1.48 (1.21)	0.53	0.58
E (mg/day)	3.12 (1.09)	3.13 (1.05)	0.94	0.86
K (mg/day)	150.42 (70.88)	154.69 (69.27)	0.66	0.59
C (mg/day)	115.17 (55.54)	115.75 (54.39)	0.94	0.93
B1 (mg/day)	2.15 (0.61)	2.15 (0.58)	0.96	0.76
B2 (mg/day)	1.75 (0.71)	1.68 (0.63)	0.46	0.42
B3 (mg/day)	27.78 (7.79)	27.36 (7.96)	0.70	0.81
B6 (mg/day)	1.37 (0.67)	1.42 (0.57)	0.58	0.39
B12 (mg/day)	3.91 (2.65)	3.32 (2.15)	0.08	0.09
B9 (μg/day)	285.77 (99.32)	295.84 (110.48)	0.49	0.29
**Other**				
Fiber (gr/day)	17.64 (5.87)	18.21 (6.26)	0.50	0.32

All data are presented as mean (±SD). *P*-values < 0.05 were considered significant. *P*-value* for adjustment model, based on energy intake.

### The correlation between MetS and its components with TMAO and KYN metabolites

According to Pearson correlation analysis, there was a positive and statistically significant correlation between TMAO levels and MetS+ (*r* = 0.176, *P* = 0.005), hypertriglyceridemia (*r* = 0.128, *P* = 0.04), abdominal obesity (*r* = 0.121, *P* = 0.05) and low HDL (*r* = 0.146, *P* = 0.02). It should be noted that despite the positive correlation between TMAO levels and hyperglycemia (*r* = 0.033, *P* = 0.601), this relationship was not statistically significant.

Moreover, there was a positive and statistically significant correlation between KYN levels and abdominal obesity (*r* = 0.128, *P* = 0.04). Also, despite the positive correlation between KYN levels and MetS+ (*r* = 0.172, *P* = 0.25), hypertriglyceridemia (*r* = 0.008, *P* = 0.90), hyperglycemia (*r* = 0.068, *P* = 0.28), hypertension (*r* = 0.022, *P* = 0.73), this correlation was not statistically significant (Table 3).

**TABLE 3:** The correlation between TMAO and KYN metabolites with MetS and its components.

Variables	TMAO (pg/mL)		KYN (nmol/L)	
	***r***	***P*-value**	***r***	***P-*value**
**Mets+**	0.176	**0.005**	0.172	0.25
**Components of MetS**				
Hypertriglyceridemia	0.128	0.04	0.008	0.90
Hyperglycemia	0.033	0.60	0.068	0.28
Abdominal obesity	0.121	**0.05**	0.12**	**0.04**
Hypertension	−0.140	0.52	0.022	0.73
Low HDL	0.146	**0.02**	−0.027	0.67

TMAO, trimethylamine N-Oxide; KYN, kynurenine; MetS, metabolic syndrome; TG, triglyceride; FBS, fasting blood sugar; WC, waist circumference; BP, blood pressure; HDL, high-density lipoprotein. *P*-values < 0.05 were considered significant, bolding in Table. *Correlation is significant at the 0.01 level (2-tailed). **Correlation is significant at the 0.05 level (2-tailed).

### The association of TMAO and KYN metabolites and odds of MetS and its components

Binary logistic regression was used, in both crude and adjusted models (for confounder potential variables such as age, energy intake, physical activity, BMI, and sex), to assess the association between TMAO and KYN metabolites and odds of MetS and its components (Table 4). In this study, we divided TMAO and KYN into two groups based on the median. Also, TMAO < 30.39 pg/mL and KYN < 297.18 nmol/L were selected as the reference group.

**TABLE 4:** The association of TMAO and KYN metabolites and odds of MetS and its components.

Variables		High levels TMAO (≥30.39 pg/mL)				High levels KYN (≥297.18 nmol/L)			
		**β**	**OR**	**95%CI**	***P*-value**	**β**	**OR**	**95%CI**	***P-*value**
**Mets+**	**Crude**	0.71	2.03	1.23–3.37	**0.006**	0.28	1.33	0.81–2.19	0.25
	**Adjusted**	0.86	2.37	1.31–4.28	**0.004**	0.39	1.48	0.83–2.63	**0.04**
**Hypertriglyceridemia**	**Crude**	0.51	1.67	1.01–2.75	**0.04**	−0.03	0.96	0.59–1.59	0.89
	**Adjusted**	0.68	1.99	1.11–3.54	**0.01**	−0.03	0.96	0.54–1.69	0.89
**Hyperglycemia**	**Crude**	0.16	1.17	0.64–2.13	0.60	0.32	1.72	0.97–2.52	0.28
	**Adjusted**	0.05	1.05	0.52–2.09	0.88	0.28	1.75	1.05–3.61	0.42
**Abdominal obesity**	**Crude**	0.48	1.63	0.98–2.69	0.19	0.51	1.68	1.01–2.77	0.06
	**Adjusted**	0.42	1.52	0.80–2.87	**0.05**	0.59	1.81	1.05–3.44	**0.04**
**Hypertension**	**Crude**	−0.22	0.80	0.40–1.58	0.52	0.11	1.12	0.57–2.21	0.73
	**Adjusted**	−0.08	0.91	0.43–1.94	0.82	0.22	1.25	0.59–2.62	0.54
**Low HDL**	**Crude**	0.65	1.92	1.09–3.38	**0.02**	−0.11	0.88	0.51–1.54	0.67
	**Adjusted**	0.67	1.95	1.04–4.06	**0.05**	−0.25	0.77	0.38–1.57	0.47

TMAO, trimethylamine N-Oxide; KYN, kynurenine; MetS, metabolic syndrome. *P*-values < 0.05 were considered significant, bolding in Table. *P*-value for adjustment model, based on age, energy intake, physical activity, BMI and sex. TMAO<30.39 pg/mL, KYN<297.18 nmol/L, MetS-, normal values of TG, FBS, WC, BP and HDL were the reference group.

In the crude model, there was a significant direct relationship between high levels of TMAO and odds MetS+ (OR = 2.03, 95% CI = 1.23–3.37, *P* = 0.006), remaining significant after adjusting for confounding variables (age, energy intake, physical activity, BMI, and sex) (OR = 2.37, 95% CI = 1.31–4.28, *P* = 0.004). Also, the odds of MetS in individuals with high levels of TMAO is 2.03 times higher than the reference group, increased to 2.37 times after adjustment. High levels of TMAO increase the odds of hypertriglyceridemia (OR = 1.67, 95% CI = 1.01–2.75, *P* = 0.04) and low HDL (OR = 1.92, 95% CI = 1.09–3.38, *P* = 0.02) in the crude model, and this association maintains even after adjusting for confounding factors (for hypertriglyceridemia: (OR = 1.99, 95% CI = 1.11–3.54, *P* = 0.01), for low HDL: (OR = 1.95, 95% CI = 1.04–4.06, *P* = 0.05). Odds of abdominal obesity were 1.63 times higher in individuals with high levels of TMAO than in the reference group, although this association was not statistically significant (OR: 1.63, 95% CI: 0.98–2.69, *P* = 0.19). After adjustment with confounding variables, despite the reduction of odds to 1.52 times compared to the reference group, it became statistically significant (OR = 1.52, 95% CI = 0.80–2.87, *P* = 0.05).

In the crude model, although the analyses showed that high levels of KYN increased the odds of Mets+ 1.33 times compared to the reference group, but was no significant statistical relationship (OR = 1.33, 95% CI = 0.81–2.19, *P* = 0.25). However, after adjustment, the odds of Mets+ have been increased to 1.48 times compared to the reference group, which is statistically significant (OR: 1.48, 95% CI: 0.83–2.63, *P* = 0.04). Additionally, there was a marginal significant between high levels of KYN and abdominal obesity (OR = 1.68, 95% CI = 1.01–2.77, *P* = 0.06), after adjusting for confounding variables, the odds of abdominal obesity in individuals with high levels of KYN were 1.81 times higher than the reference group (*P* = 0.04).

## Discussion

To the best of our knowledge, this survey is the first study that investigated two types of metabolites obtained from gut microbiome (Serum KYN and TMAO) with MetS and its components in the Iranian population. The present study showed that there is a positive correlation between TMAO and MetS. In the findings of our study, it was observed that high levels of TMAO can increase the odds of hypertriglyceridemia and abdominal obesity and can have a reducing effect on low HDL. In the case of KYN, the odds of MetS could be increased after adjusting for confounders, in addition, individuals with high levels of KYN were more likely to have abdominal obesity. As one of the basic risk factors for cardiovascular diseases and all-cause mortality, MetS plays an important role in people’s health (36). Despite its widespread in different countries, including America, as well as its rising graph in Iran, it has attracted a lot of attention in recent years (36–38). The findings of the present study showed that people with MetS had higher DBP, TG, LDL, TC, FBS, WHR, WC, and HC than people without MetS. People with MetS also had lower HDL than the other group without this disorder. In another study, people with MetS had higher BMI, DBP, TG, SBP, body weight, FBS, WC, and lower HDL compared to healthy people(39). Today, studies are reporting on the role of metabolites obtained from the gut microbiome in the pathogenesis of MetS, and TMAO and KYN are among the metabolites investigated in this study (6, 7).

TMAO is a metabolite that is produced by the microbial metabolism of choline from animal food sources and L-arginine from meat. The L-arginine metabolism pathway leads to the production of two other intermediate metabolites, namely crotonobetaine, and γ-butyrobetaine, which ultimately cause TMAO (40). In the present study, TMAO levels in people with MetS were about 51.49 (pg/mL), and in people without this disease, it was 37.50 (pg/mL). In another study, the circulating levels of TMAO in people with MetS were about 10.65 ± 1.62 (μM), and in people without this disease, it was reported as 6.82 ± 3.17 (μM) (11). In our study, we observed that levels higher than 30.39 pg/mL could increase the odds of MetS. In the other study, the acceptable cut-offs for circulating levels of TMAO for predicting the presence of MetS was ≥ 8.74 μM (11). (The reason for the different expression of the measurement units is the use of various kits in the measurement of TMAO and KYN and as the level of TMAO depends on various factors including age, gender, race species of the microbiome, and food intake, maybe the level of TMAO in our study was low). In the case of TMAO, there was a significant positive correlation between high levels of TMAO and MetS, which increased odds after adjusting for confounders, and also, there was a positive correlation between TMAO and abdominal obesity and also with low HDL. The studies conducted on TMAO reveal the destructive effects of this metabolite, such as the formation of macrophage foam cells (41), endothelial dysfunction (42), vascular inflammation and activation of inflammation (43–46), increased platelet reaction and thrombosis (47, 48), and reduced cholesterol reverse transport (49). Meanwhile, diet plays a key role in TMAO production. In a study conducted in 2023 by Meng Wang et al., more meat consumption can increase the risk of atherosclerotic cardiovascular disease through increasing metalloids such as TMAO (40). According to the studies conducted on TMAO, this metabolite can cause insulin resistance by blocking the hepatic insulin signaling pathway and also by causing inflammation in adipose tissue in mice, which are themselves one of the components of MetS (50). Meanwhile, insulin signaling suppresses flavin-containing monooxygenase 3 (FMO3), which is the enzyme that produces TMAO (51). So in people with insulin resistance or obese people, the level of the FMO3 enzyme increases(51, 52). Moreover, FMO3 can induce the production of FoxO1, which is responsible for regulating some gluconeogenic genes and as a result, adjusting glucose production in the liver. The findings indicate that the suppression of FMO3 through the inhibition of FoxO1 can prevent blood fat, hyperglycemia, and atherosclerosis (51). According to the studies, high levels of TMAO can increase lipid metabolism disorders and inflammatory response (43, 47, 50). Based on the evidence, TMAO can be a factor in the occurrence of obesity as well as the basis of obesity-related disorders such as body weight, BMI, and visceral fat index (VAI), and VAI itself can reduce insulin sensitivity (11, 52–54). With the help of new findings, TMAO is considered as a prospective indicator for detecting MetS (11). The evidence of the present study shows that there was a positive and statistically significant correlation between TMAO levels and hypertriglyceridemia. In a study conducted by Lin Ding et al in 2018, mice with the apoE genotype were fed a diet containing TMAO for 8 weeks. The result of the study was the fat in the serum of mice, and the possibility of inhibiting the synthesis of hepatic bile acid is considered the reason for this increase in fat (55).

Kynurenine is a metabolite that is created in the human body in 90% of the way of metabolizing TRP and can be directly or indirectly affected by factors such as diet and gut microbiota (10). Diet can alter KYN levels in the body. In the study conducted by Sandra Tillmann and her colleagues in 2021, it was shown that a methyl-deficient diet can regulate the KYN pathway (56). From other studies in this field, the relationship between the ketogenic diet, ginseng polysaccharides intake, a diet with limited calories, and a high-fat diet has been reported (57–59). In this study, the relationship of KYN levels obtained from serum on MetS and its components was investigated. The findings of the present study show that high levels of KYN increase the odds of MetS. There are not many studies on the relationship between KYN and MetS and its components. The relationship between inflammation and depression is not hidden from anyone and many studies have shown the relationship between these two disorders (60–63), but in the meantime, KYN also plays its role under the influence of inflammation (64). In the study published in 2010 by Gregory F et al., despite considering inflammation as one of the important factors of MetS, it reveals the role of inflammation on KYN (64). So inflammation can change the pathway of TRP synthesis to KYN production by inducing the transcription of one of the rate-limiting enzymes of the TRP /KYN pathway, called indoleamine 2,3-dioxygenase (IDO). Furthermore, KYN can cause the development of MetS through neurotoxic and prooxidative, apoptotic effects as well as positive regulation of phospholipase A2, prostaglandin, 5-lipoxygenase, arachidonic acid, nitric oxide synthase, and leukotriene cascade (64). picolinic acid and quinolinic acid are two intermediary metabolites in the metabolism of KYN, and the increase of KYN causes the increase of these two metabolites. These metabolites can increase lipid peroxidation through the stimulation of nitric oxide synthase (NOS) and the mediation of 3-hydroxy anthranilic and 3-hydroxykynurenine acids, and as a result, through the stimulation of arachidonic acid release, the activation of inflammatory factors such as leukotrienes, through the activation of arachidonate 5-lipoxygenase (5-LOX) and prostaglandins, through the active action of cyclooxygenase (COX) (Melillo et al., (65); Oxenkrug, (66)). Xanthurenic acid is another metabolite in the KYN-nicotinamide adenine dinucleotide pathway that creates an antigenic compound unrecognizable from insulin by reacting with insulin and eventually causes insulin resistance by reducing insulin sensitivity (67–70). According to studies, IDO has been reported to be inversely related to high-density lipoprotein and directly related to C-reactive protein (71, 72). The present study shows that there is a correlation between KYN levels and abdominal obesity. In the study conducted by Judith A. Finkelstein et al., free TRY was decreased in the blood of obese mice (73). Harald Mangge and colleagues showed that there is a relationship between KYN/TRP and MetS, BMI, and abdominal obesity, so that the higher this ratio, the more relationship was observed, and also MetS and this ratio both affect each other (74). In a study conducted on people with HIV in 2020, the ratio of quinolinic acid to kynurenic acid increased due to abdominal obesity, which means that the pro-inflammatory pathway of KYN metabolism is activated and the result of this pathway can increase systemic inflammation and decrease anti-inflammatory molecules (75).

### Limitations

Finally, we acknowledge several limitations that need to be considered for this study, mainly resulting from being a case-control study design that can not preclude causal inferences from the Serum KYN and TMAO Levels novel biomarkers associated with MetS and its components in adults. Secondly, to investigate the metabolites obtained from the gut in this research, metabolites were measured in serum, and serum factors can be affected by many different conditions of a person. Thirdly, although an acceptable number of confounders were controlled in this study, the presence of residual confounders cannot be denied. Fourthly, investigations with a larger sample size must evaluate this association.

### Strengths

This study used novel biomarkers for assessing the Serum KYN and TMAO Levels which is a new approach and consists of a new view and it is the first study that examines these two biomarkers simultaneously. This study is based on a case-control study that was investigated on two groups of MetS. This is the first study that evaluates Serum KYN and TMAO with MetS along with its components. A wide range of confounding factors have been taken into account to achieve reliable results.

## Conclusion

The result of this investigation shows that there was a positive correlation between TMAO and MetS and its components. Also, we observed a positive correlation between KYN and abdominal obesity. Also, our study showed that high levels of TMAO (≥30.39 pg/mL) can increase the odds of MetS, hypertriglyceridemia, abdominal obesity, and low HDL. Moreover, high levels of KYN (≥297.18 nmol/L) can raise the odds of MetS and abdominal obesity. Therefore, the metabolites obtained from the gut microbiome can have effects on MetS and the risk of cardiovascular diseases, and it is very important to pay more attention to these metabolites with a larger sample size in the future.

## Data availability statement

The original contributions presented in the study are included in the article/supplementary material, further inquiries can be directed to the corresponding author.

## Ethics statement

The studies involving humans were approved by TUMS evaluated and approved the study protocol (IR.TUMS.MEDICINE.REC.1401.064). The studies were conducted in accordance with the local legislation and institutional requirements. The participants provided their written informed consent to participate in this study. Written informed consent was obtained from the individual(s) for the publication of any potentially identifiable images or data included in this article.

## Author contributions

AM: Writing – original draft. MM: Writing – original draft. AK: Writing – original draft. HA: Writing – original draft. FA: Formal analysis, Writing – original draft. NS: Writing – original draft. MH: Writing – original draft. MR: Supervision, Writing – original draft. PK: Writing – review and editing. KM: Supervision, Writing – review and editing.
